# Dual-Family Peptidylprolyl Isomerases (Immunophilins) of Select Monocellular Organisms

**DOI:** 10.3390/biom8040148

**Published:** 2018-11-15

**Authors:** Sailen Barik

**Affiliations:** 3780 Pelham Drive, Mobile, AL 36619, USA; barikfamily@gmail.com; Tel.: +1-251-454-1255

**Keywords:** peptidylprolyl isomerase, chaperone, immunophilin, extremophile, intrinsic disorder

## Abstract

The dual-family peptidylprolyl *cis-trans* isomerases (immunophilins) represent a naturally occurring chimera of the classical FK506-binding protein (FKBP) and cyclophilin (CYN), connected by a flexible linker. They are found exclusively in monocellular organisms. The modular builds of these molecules represent two distinct types: CYN-(linker)-FKBP and FKBP-3TPR (tetratricopeptide repeat)-CYN. Abbreviated respectively as CFBP and FCBP, the two classes also exhibit distinct organism preference, the CFBP being found in prokaryotes, and the FCBP in eukaryotes. This review summarizes the mystery of these unique class of prolyl isomerases, focusing on their host organisms, potential physiological role, and likely routes of evolution.

## 1. Introduction

To start with, a quick note on nomenclature used in this review is in order. The two classic immunophilin families are cyclophilin and the FK506-binding protein, traditionally abbreviated as CYP and FKBP, respectively [1,2]. Although the cyclophilins are often given the acronym CYP, I have used the recently accepted root, CYN [3]. The terms “immunophilin” and “peptidylprolyl *cis-trans* isomerase” (PPI or PPIase) have often been used interchangeably, since both are rooted in the early discovery that these proteins specifically bind the immunosuppressive drugs cyclosporin (e.g., cyclosporin A or CsA) and FK506 (also known as Tacrolimus), respectively, and also catalyze *cis-trans* isomerization of the peptidylprolyl bonds in proteins [4,5,6]. Though the binding generally leads to inhibition of the PPIase activity, the immunosuppressive function of these drugs is in fact due to the ability of the drug-PPI complexes to inhibit key signaling proteins. Specifically, both CsA-CYN and FK506-FKBP complexes, in spite of the highly dissimilar sequences of CYN and FKBP, bind to and inhibit calcineurin (a Ca^+2^-dependent protein phosphatase); this reduces the dephosphorylation of NF-AT (nuclear factor of activated T-cells), a calcineurin substrate and a transcription factor [7,8]. In T-cells, activation of the T-cell receptor increases intracellular calcium, which activates calcineurin. The activated calcineurin dephosphorylates NF-AT, which then moves to the T-cell nucleus and increases the transcription of genes for interleukin (IL)-2 and other cytokines, triggering immune response [8]. Inhibition of the dephosphorylation of NF-AT thus leads to reduced effector T-cell function, and hence immunosuppression [8]. Another immunosuppressive drug, rapamycin, also binds FKBP; however, instead of inhibiting calcineurin, the rapamycin-FKBP complex inhibits TOR (‘target of rapamycin’), a protein kinase important for T-cell proliferation [9]. It is important to realize that drug-binding is important for its clinical significance, but is not encountered in the natural environment of the untreated cell [1,2]. A common physiological function, attributed to both classes of PPI, is their role in protein folding, whereby they act as chaperones and co-chaperones to catalyze the proper folding of a variety of client proteins [1,2]. Although much of the chaperone function is due to *cis-trans* isomerization of the proline peptide bonds in the client protein, catalyzed by the peptidylprolyl isomerase activity [10,11], there are important examples in which these proteins function as scaffolds, independently of their PPIase activity, such as refolding of arginine kinase by cyclophilin A [12], FKBP51-mediated activation of IKK (IκB kinase; the kinase complex that is required for NF-κB activation [13]), and modulation of human telomerase activity by FKBP51 and FKBP52 [14,15].

While the CYN and FKBP families have been known and studied extensively for about three decades, the dual-family peptidylprolyl isomerases (abbreviated here as DFPPIs) were not known until 2005, when the first member of the FCBP family was reported in *Toxoplasma gondii* (*T. gondii*, abbreviated Tg), a monocellular protozoan parasite, and the first CYN-(linker)-FKBPs (CFBPs) were reported in Flavobacteria and Spirochete [16]. Studies of the recombinant TgFCBP and one of the flavobacterial CFBPs demonstrated their overall PPIase activity, as well as the PPIase activity of individual CYN and FKBP domains, which could be inhibited by CsA and FK506, respectively [16]. Both drugs also inhibited *T. gondii* replication, suggesting a physiologically important role of FCBP in this parasite. The full-length DFPPIs as well as the individually expressed CYN and FKBP domains were able to promote refolding of a model substrate protein in vitro, suggesting the DFPPIs are in fact dual-family chaperones [16]. In the years that followed, sequence homology search unraveled several dozen more FCBP and CFBP in the newly sequenced genomes [17], although their biochemical properties were not studied. In this review, I collectively refer to these CFBP and FCBP sequences as DFPPIs, and summarize our current knowledge on these proteins along with a broader view of their potential function.

More specialized reviews of DFPPI subsets and recent bioinformatic analysis of their structure can be found elsewhere [17,18,19].

## 2. Dual-Family Peptidylprolyl Isomerase Structure

**Figure 1:**
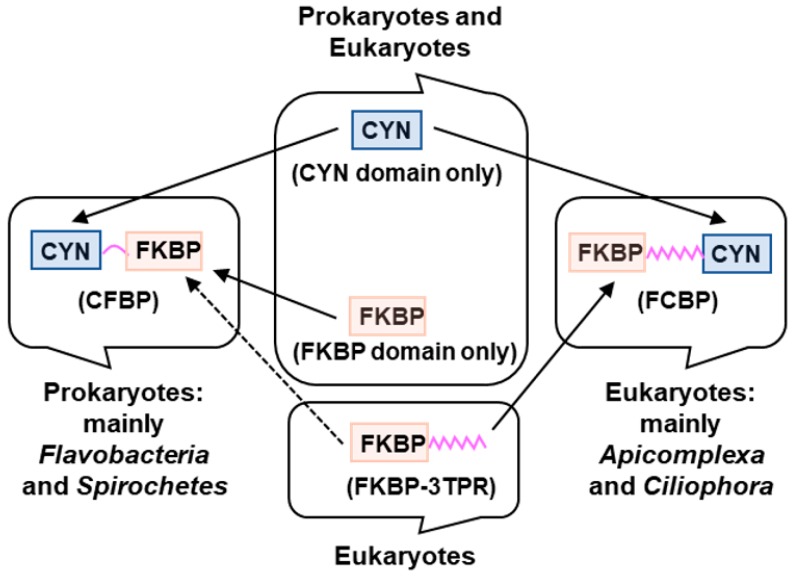
Proposed biogenesis of the two types of dual-family PPIases (DFPPIs), detailed in the text. CYN: cyclophilin; FKBP: FK506-binding protein; TPR: tetratricopeptide repeat. The known occurrences of the domains, namely CYN (cyan), FKBP (salmon), TPR (pink), in the various organisms are indicated. The same color code has been used in all Figures of this review. Of note, all DFPPI-containing organisms, found so far, are monocellular.

Comprehensive survey and phylogenetic analysis of all currently available DFPPIs have documented several specific distinctions between the CFBP and the FCBP classes (Figure 1).

**Figure 2:**
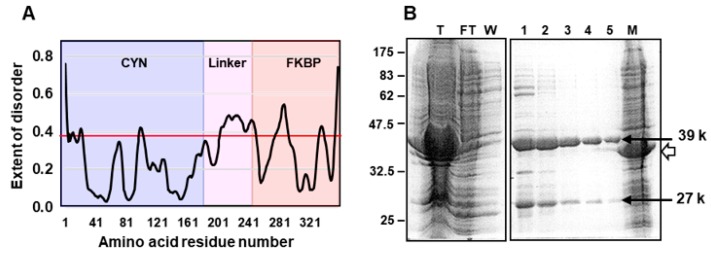
(**A**) Intrinsic disorder in the linker peptide region (the central pink area), connecting the CYN and FKBP domains in FjCFBP39 of *Flavobacterium johnsoniae* (GenBank WP_012024434.1), computed with PrDOS (prdos.hgc.jp), as previously described [19]. The red horizontal line is the baseline for disorder cutoff, set at 10% false-positive rate. Another small peak above this line (around residue 290), is a short loop region, visible and conserved in the FKBP crystal structures, and thus, not considered “intrinsically disordered” [19]. The domain areas are color-coded as in Figure 1: CYN, cyan; linker, pink; FKBP, salmon. (**B**) Fragmentation of FjCFBP39 during purification. The coding sequence of FjFCBP39 was cloned with N-terminal His-tag in pET15b plasmid, and expressed in *Escherichia coli* BL21 (DE3) pLysS, as described before [16]. Attempts were made to purify the recombinant protein by standard Ni^+2^-agarose affinity chromatography [16] (Novagen Inc, Madison, WI, USA) and fractions at various steps of purification were analyzed by sodium dodecyl sulfate-polyacrylamide gel electrophoresis (SDS-PAGE), followed by staining with Coomassie Blue. Protein molecular weight markers of the indicated Mr (relative mobility) are shown at left. The prospective bands of the full-length protein of the structure His-CYN-linker-FKBP and Mr 39 k, and its putative His-CYN fragment (following cleavage within the linker) of 27 k are indicated with arrows. The white arrowhead points to a 34 k band (lane M), corresponding to another, irrelevant recombinant protein in total bacterial extract, used as molecular weight marker. The other lanes are as follows: T, total bacterial lysate containing FjFCBP39; FT, column flow-through (unbound proteins); W, wash (to remove unbound proteins); lanes 1–5, gradual fractions eluted with 0.5 M imidazole buffer, as recommended by the manufacturer. Note the presence of approximately equimolar amounts of the 39 k and 27 k bands in all fractions. The FKBP part does not bind to the column because it is at the C-terminus, lacking His-tag.

In terms of structure, all FCBPs contain a 3TPR domain, linking the FKBP and the CYN domains. The TPR (tetratricopeptide repeat) is a degenerate repeat motif, roughly 34-amino acid long, which is structurally flexible because it is essentially all alpha helices joined by unstructured loops. Multiple tandem repeats of TPR can be found in a wide variety of proteins, in which they participate in protein-protein interactions. All FCBPs contain three nonidentical repeats of TPR, referred as 3TPR [17]. In sharp contrast, the CFBPs do not contain TPR, but instead uses short peptide sequences, averaging ~50 amino acids in length, to serve as a linker between the CYN and FKBP domains [16]. Bioinformatic analysis showed that all CFBP linkers, in spite of their sequence dissimilarity, possess intrinsic disorder (ID), a hallmark of flexible protein linkers [19], as shown here with *Flavobacterium johnsoniae* CFBP (Figure 2). Thus, both classes of DFPPIs contain flexible linkers, serving as connectors between the two classical PPI/immunophilin domains, which offered the first indication that the two domains may function as independent functional modules, able to freely bend against each other [17,19]. Of note, there are at least two other PPIase families, namely the bacterial parvulin family (and its distant eukaryotic homolog, Pin1), and the bacterial trigger factors [3]. However, they are entirely different in sequence from both the cyclophilin and the FKBP families, as well as from the DFPPIs, and therefore, are not considered here further.

**Figure 3:**
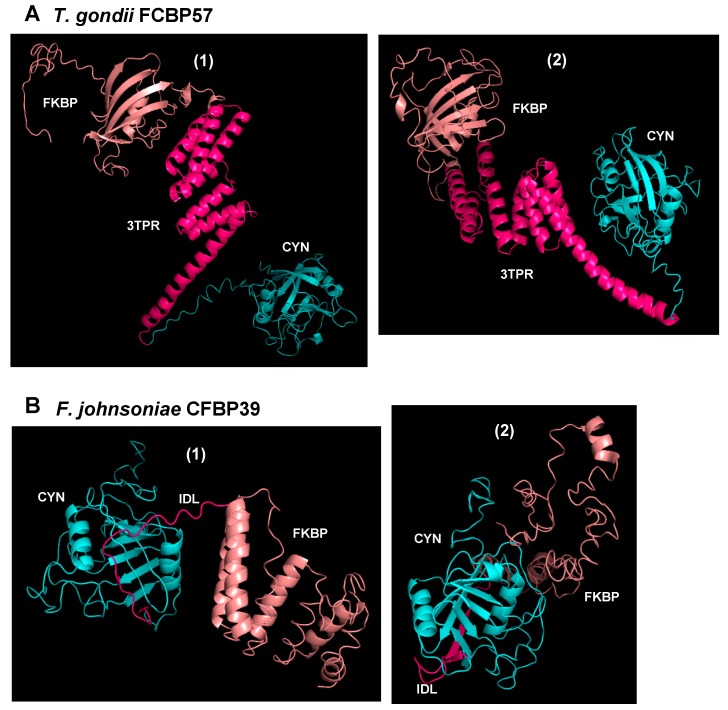
Homology-based modelling of three-dimensional structures of representative (**A**) FKBP-3TPR-CYN (FCBP) and (**B**) CYN-(linker)-FKBP (CFBP) proteins, deduced by I-TASSER and drawn with PyMOL (https://pymol.org, Schrödinger, LLC, USA), as described before [17]. As indicated, the proteins are from *Toxoplasma gondii* and *Flavobacterium johnsoniae*, with theoretical molecular mass of 57 kDa and 39 kDa, respectively. The domains are color-coded: CYN domain, cyan; 3TPR linker in *Toxoplasma gondii* FCBP57 (TgFCBP57) or the intrinsically disordered linker (IDL) in FjCFBP39 (see Figure 2), pink; FKBP domain, salmon color. As seen, the flexibility of the linkers in both polypeptides clearly allowed large changes in the relative orientation between the CYN and FKBP domains, and two arbitrarily chosen conformers (numbered 1 and 2) are presented here.

The three-dimensional structure of DFPPIs remains unsolved, and our multiple attempts to crystallize TgFCBP and the 39 kDa *F. johnsoniae* CFBP have failed, most likely due to the conformational flexibility of the linker regions [20]. Nonetheless, advanced 3D simulation with the I-TASSER server (https://zhanglab.ccmb.med.umich.edu/I-TASSER), using the known crystal structures of similar CYN and FKBP sequences, yielded the expected structures, i.e., the CYN and FKBP domains, linked with TPR or the short linker with ID [17]. Interestingly, the simulations actually yielded an ensemble of multiple structural conformers in which the two domains were oriented at various degrees relative to each other, clearly using the flexible linker as hinge (Figure 3).

Lastly, ID regions are known for their susceptibility to proteolysis, which is often used as diagnostic of such regions [21,22]. Although controlled proteolysis studies have not yet been conducted on any DFPPI, we have consistently observed, during the early steps of purification of recombinant FjCFBP39, polypeptide fragments that are shorter than the full-length protein and coincide in size with proteolytic cleavage within the ID linker (Figure 2).

## 3. Organisms Harboring Dual-Family Peptidylprolyl Isomerases

An enigmatic aspect of the DFPPIs is their absence in all metazoan (multicellular) organisms, such as higher eukaryotes, including vertebrates. Comprehensive survey and phylogenetic analysis of all currently available DFPPIs showed that the CFBPs occur exclusively in prokaryotes and the FCBPs, exclusively in monocellular eukaryotes, with no exceptions found so far [17]. It is also equally enigmatic that all DFPPIs are limited to a few genera; in fact, only a little over 300 DFPPI genes could be found collectively in all genome databases (Table 1).

In other words, only a miniscule fraction of organisms in the living world contain DFPPIs. As summarized (Table 1), out of 278 CFBP sequences, an overwhelming number (235) occur in Flavobacteria (currently placed under the extended *Bacteroides* family, while smaller numbers occur in other bacteria, namely the spirochetes and the proteobacteria. Similarly, out of a total of 40 FCBP sequences found in monocellular eukaryotes, 21 occur in the protozoa of the *Apicomplexan* family and the related *Ciliophora*, whereas the rest are split among the oomycetes and the diatoms, only two sequences being found in the dinoflagellates. Also notable is the total absence of DFPPIs in Archea [17], the third kingdom of life, considered distinct from prokaryotes and eukaryotes. Our initial expectation that the DFPPIs may be highly prevalent in the Archea, both being unique in nature, thus met with disappointment.

**Figure 4:**
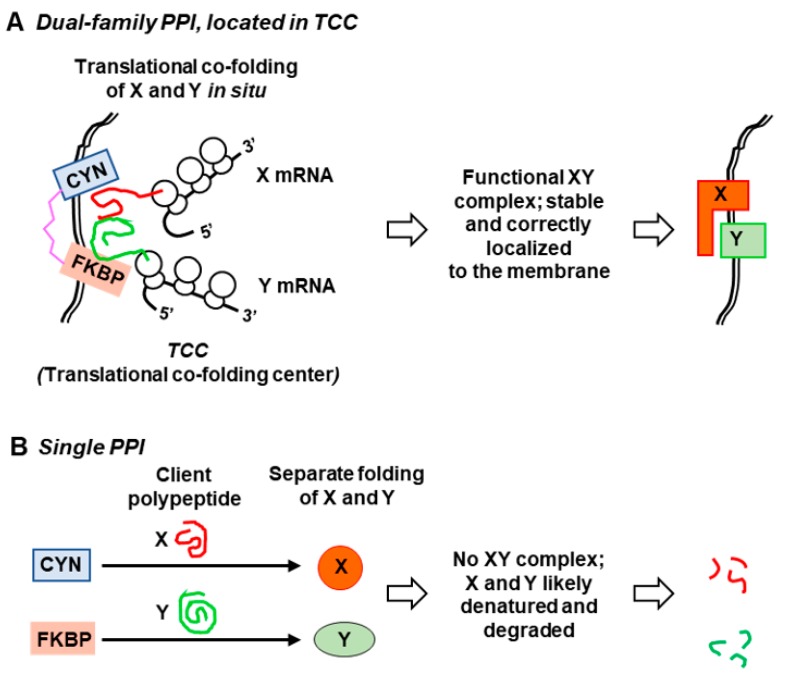
Proposed model for co-folding of multiple polypeptides by dual-family peptidylprolyl isomerase (PPI) in a “translational co-folding center” (TCC). The domain color codes are the same as in Figure 2 and Figure 3: CYN, cyan; linker, pink; FKBP, salmon. In this model, (**A**) the assembly of specific multisubunit complexes is facilitated by joint folding (co-folding) of multiple nascent subunits (X and Y, in red and green, respectively) in situ. It is efficiently achieved by the DFPPI, localized in strategic places, such as cellular or organellar membranes (shown as a double line). Polyribosomes translating the mRNAs for X and Y may also reside at these sites to efficiently deliver the nascent polypeptides to the DFPPI, hence the proposed name, translational co-folding center. The chimeric PPI domains of DFPPI act in cis, and the flexible hinge allows simultaneous accommodation of multiple and diverse clients. The proximity of the TCC to the membrane also allows rapid membrane localization of the XY complex in situ. The kinetics of such co-folding and localization can be critically important in high-stress, extreme environments of the DFPPI organisms, such that inefficient co-folding by single PPIs (CYN or FKBP) (**B**) would rapidly denature and degrade X and Y.

We conjectured that the biological role of DFPPIs could be speculated from the environmental conditions at which of the parent organisms persist and proliferate. Although there is no single trait that is common to all DFPPI-harboring organisms, several areas of their habitat and physiology are highly similar, as previously detailed [17]. In brief, the DFPPI organisms in general possess exotic lifestyles and are extremophiles, such as halophilic, psychrophilic, thermophilic, acidophilic, and sulfur-reducing. Many have been isolated from marine waters with high salt content and extremely low organic nutrient concentration. Several organisms, most notably the Apicomplexan protozoa, such as *T. gondii*, are obligatory parasites that must survive in the body fluids of the host animal and evade the immune response. In many such situations, mobility is an asset, and it is perhaps no coincidence that essentially all DFPPI organisms exhibit some form of motility and locomotion. This realization has led to the postulate [16,17,19] that the double-pronged chaperone function of DFFPI is needed for simultaneous and efficient folding of multisubunit motor and transporter complexes, especially in extreme conditions, as depicted in the model (Figure 4).

## 4. Physiological Function of the Dual-Family Peptidylprolyl Isomerases

Although biochemical studies of recombinant DFPPIs as described before [16] documented their PPIase activity and chaperone function, their physiological role in the organism is largely unknown. In *T. gondii* cells, the FCBP most likely functions as a PPIase, as both CsA and FK506 inhibited replication of the parasite in culture, measured by parasitic DNA synthesis [16]. The *T. gondii* genome encodes multiple CYN paralogs, and therefore, it could not be ruled out that the antiparasitic effect of CsA is due to inhibition of the CYNs. However, the antiparasitic effect of FK506 must due to inhibition of the FKBP domain of TgFCBP, as there is no other FKBP sequence in the parasite genome [16]. In analogy to the single family PPIases, it is safe to assume that the DFPPIs play a major role to facilitate folding of their client proteins, which also remain unidentified. Expanding on our previous suggestions [16,17,19], I have proposed a model (Figure 4), in which the bifunctional DFPPI is located, perhaps together with translating polyribosomes (polysomes), at specialized cellular sites that is tentatively named “translational co-folding centers” or TCCs.

The TCC model (Figure 4) embodies the following features for the proper folding and assembly of nonglycosylated proteins: (1) the polysomes deliver the nascent client polypeptides directly to the DFPPI in situ; (2) the DFPPI then promotes simultaneous folding of multiple polypeptides, using both the CYN and FKBP modules, which would not happen if each subunit were to be folded separately by single-family chaperones; (3) the flexible connecting peptide (loop or TPR) facilitates hinged movement of the two PPIase modules, allowing accommodation of diverse client proteins at various spatial orientations, as needed for joint chaperoning and assembly; (4) location of the TCC at the proper site allows prompt delivery of the assembled complex; for example, location at or near a membrane would allow coupling of co-translational folding with incorporation into the membrane. In FCBP-harboring monocellular eukaryotes, such as *T. gondii*, the glycosylated proteins are processed and transported through the endoplasmic reticulum, and delivered to the membranous structures, essentially as in higher eukaryotes [23]; thus, in these organisms, the FCBP-containing TCC would be more important for a subset of protein complexes or secreted proteins that are nonglycosylated. The CFBP, on the other hand, belong to bacteria, which lack glycosylation, and hence may be involved in chaperoning a larger repertoire of membrane proteins through the TCC. Both types of DFPPI may also be important for cytoplasmic protein complexes that need to be assembled near the membrane; (e) the high efficiency and speed of the overall process is particularly important in extreme environments, such as high heat or low temperature, in which many DFPPI organisms thrive in nature [17]. Without rapid folding, the unfolded and unassembled proteins, are likely be denatured and degraded.

As argued previously [17], the need for simultaneous multisubunit chaperoning may be particularly acute for membrane-anchored complexes, which must also be vectorially transported into the membrane in correct orientation. A common feature of many DFPPI organisms is various forms of monocellular motility, such as swimming and gliding, which are essential for their survival and are guided by membrane-anchored motors such as flagella and other appendages [17,24,25,26]. Thus, these motor complexes may be particularly dependent on DFPPI-mediated chaperoning. The same can be true for membrane channels and transporters such as the ATP-binding cassette (ABC) transporter, which are also large multisubunit complexes [17].

## 5. Available Evidence for the Translational Co-Folding Center Model

It should be noted that DFPPI is a relatively recent discovery (in comparison to the single PPIs), and besides the biochemical properties of *T. gondii* FCBP reported in the original paper [16], no focused studies have been conducted on any DFPPI. The physiological substrates of DFPPI also remain unknown. Overall, there is a dearth of experimental data on the natural role DFPPI, and hence, direct evidence for the complete TCC model of DFPPI function is lacking. Nevertheless, several lines of evidence support many individual parts of the model, which will be pieced together here.

**Figure 5:**
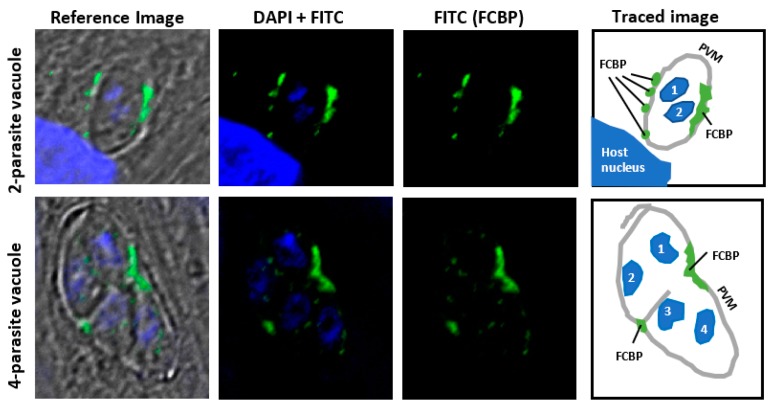
Membrane location of *Toxoplasma gondii* FCBP (TgFCBP). Human foreskin fibroblast (HFF) cells were grown in monolayer on coverslips, and asynchronously infected with *T. gondii* RH strain as described previously [16]. Infected cells were fixed and immuno-stained with primary antibody against the peptide CGSGSDKVDVDPASIVD (TgFCBP residue 18–34), described before [16], followed by specific fluorescein isothiocyanate (FITC)-conjugated secondary antibody. Images were captured in a Nikon TE2000 microscope (Nikon Instruments Inc, Melville, NY, USA). The stains were: 4′,6-diamidino-2-phenylindole (DAPI; blue) for nucleus; FITC (green) for FCBP. The reference image is a merger of both colors on phase-contrast bright-field image. The diagram on the right was generated by manually tracing the areas of interest using the Microsoft PowerPoint free-drawing tool (Microsoft Co., Redmond, WA, USA). Two representative fields, respectively containing a two-cell and a four-cell parasite stage, are shown as numbered by the nuclear DAPI stain. *T. gondii* replicates in the host cell cytoplasm inside the parasitophorous vacuole (PV), surrounded by its membrane (PVM), as shown. The PV is often found close to the host cell nucleus, a portion of which is seen as the large DAPI-stained area in the two-parasite cell image. Note that the FCBP (green) is most abundant on or near the PVM, as shown in the tracing, whereas the interior of the PV contains only a few small spots. (Image courtesy: Dr. Joel Andrews).

The potential membrane location of TCC is suggested by indirect immunofluorescence staining of *T. gondii* FCBP (Figure 5). An obligatory parasite, *T. gondii* readily infects nucleated mammalian cells and creates a unique structure, known as the “parasitophorous vacuole” (PV), which is separated from the host cell cytoplasm by a semipermeable membrane (PVM), primarily made of cell membrane in which parasitic proteins are later incorporated [27]. The parasite replicates inside the PV by “schizogony”, producing a net doubling of the parasite cells in each division cycle. The PVM plays an essential role in parasite growth by protecting the parasite from the host’s innate immunity while allowing uptake of nutrients and exchange of other small molecules [27]. Immunofluorescence studies using a highly specific peptide antibody (Appendix A) revealed that the TgFCBP was located predominantly on or near the PVM (Figure 5).

The number or location of the FCBP spots did not coincide with those of the parasites (Figure 5), suggesting that the TCCs in this case serve in a shared role for all sister parasites growing inside the PV. By being associated with PVM, the FCBP can facilitate the assembly, not only of proteins that are integral to the PVM but also those that associate with the cytoplasmic face of the PVM. The former class may include various transporters, while the latter class is exemplified by the “dense granular proteins” (GRA proteins) of the parasite, such as GRA3, which is a soluble secretory protein but forms a large oligomer that resides in the PVM and the intravacuolar network, where it may function as a pore-forming molecule [28,29].

The negative effect of extreme physical and chemical parameters such as heat, cold, or high acidity, on the thermodynamic and kinetic stability of proteins is well known [30,31,32,33,34], and as stated before, many DFPPI organisms live in such extreme environments [17]. There is ample evidence that binding of partner subunits, and even small molecule ligands, cofactors and ions, often stabilize a protein from denaturation and proteasomal degradation [35,36,37,38]. It is also known that molecular chaperones from bacteria to man protect cellular proteins from denaturation under stress conditions [39,40,41,42]. It is, therefore, quite likely that the DFPPIs mediate a similar effect by facilitating oligomerization. In this role, the DFPPIs can be regarded as “assembly” chaperones, a name that was recently proposed for specialized proteins that facilitate ribosomal incorporation of specific ribosomal proteins, such as Acl4, which serves a dual function to facilitate nuclear import and simultaneously protect unassembled RpL4 from the cellular degradation machinery [43].

Lastly, many DFPPI organisms exhibit gliding or swimming motility that makes use of specialized cilia or flagella [23,24], likely essential for survival and nutrient scavenging in the wild (see below). The early stages of flagellar biosynthesis in bacteria requires the proper and concerted positioning of multiple proteins in the flagellar architecture [44]. The complexity of the process can be appreciated by the fact that the membrane ring alone is composed of 25 copies of the protein flagellar motor protein F (FliF) [45]. Furthermore, a seminal recent study presented direct evidence that co-folding of two filament ring protein (‘Fli’ protein) domains forms the basis of membrane and cytoplasmic ring interface within the flagellar motor [46]. It is imperative that such co-folding events, whether homo- or hetero-oligomeric, will be vulnerable to denaturing conditions, and thus, highly benefit from in situ co-folding.

Although the flagella of the DFPPI organisms have not been dissected in molecular detail, there is an impressive body of literature of the gliding motility of *Flavobacterium johnsoniae*, which harbors a CFBP. A prototype member of the Bacteroides family, *F. johnsoniae* exhibits robust gliding, which requires several genes of the “Gld” loci [47,48,49]. Interestingly, while the CFBP is encoded by *GldI* gene [16], several other *Gld* genes express the subunits of a putative ABC transporter, a multi-subunit complex [47,48]. Yet other *Gld* genes encode proteins that form the Por secretion system (PorSS), suggesting that the secretion system and motility may be intimately related [50,51]. In fact, several DFPPI bacteria, belonging to the *Bacteroidetes* phylum, possess homologs of the *Gld* and the *PorSS* genes [50]. Together, the bacterial results are consistent with the premise that a membrane-anchored, DFPPI-containing TCC would be ideally positioned to assist in the assembly of the motility apparatus as well as membrane transporters.

## 6. Phylogenetic Origin of the Dual-Family Peptidylprolyl Isomerases

Perhaps the single biggest mystery of the DFPPIs is their rarity as well as sporadic distribution, for which no clear explanation currently exists. Our extensive searches have revealed that they are present only in a tiny fraction of organisms of the living world (Table 1). Even when they are present in a family of organisms, they do not occur in all members of that family. While a large number of flavobacteria possess CFBP, many do not, although they may contain single CYN homologs. For example, several species of the *Psychroflexus* genus encode CFBP (e.g., WP_019038212.1 of *P. tropicus*) [17], but *P. salarius* has none, although it has single CYN homologs (e.g., WP_083574495.1). Besides Toxoplasma, FCBP occurs in several other Apicomplexan parasites, but notably, not in plasmodia, the parasite that causes malaria [17]. In the *Eimeria* genus of Apicomplexan parasites, *E. brunetti* (CDJ53046.1), *E. maxima* (XP_013335079.1) and *E. tenella* (AET50588.1) have one FCBP each [17], whereas *E. falciformis* has none [52]. It should be noted that most Apicomplexa belong to one of the three types, namely: gregarines, coccidians (*Eimeria*, *Toxoplasma*) and haemosporidians (*Plasmodia*, *Babesia*, *Theileria*). Interestingly, the FCBPs are found in the two latter types [17], not in the gregarines. The reason behind such differences within a subset of organisms can only be speculated. Comparative genome analysis [52], for example, have shown that *E. falciformis* is missing important virulence factors that are present in *T. gondii*. The *E. falciformis* genome also possesses a reduced set of transmembrane transporters, suggesting the existence of an altered mode of iron uptake. It is possible that such divergence underlie the differential need for FCBP in different *Eimeria* species.

It is also intriguing that several DFPPI species contain multiple DFPPI paralogs that are nonidentical in sequence; for instance, *Flavobacterium johnsoniae* has three CFBPs (WP_012024258.1, WP_012024433.1, WP_012024434.1), and so does *Flavobacterium psychrophilum* (WP_034098691.1, YP_001296758.1, YP_001296759.1), while *Paramecium tetraurelia* (a ciliophora) encodes two (XP_001444554.1, XP_004031957.1). In general, there is no consistent correlation between the number of DFPPIs and single PPIs in an organism, as shown here with two class-representative DFPPI organisms (Table 2). For example, *T. gondii* contains a single FCBP, no FKBP, and 14 CYNs (Table 2), whereas *Hammondia hammondi*, a highly similar parasite, contains one FCBP, two FKBPs and 12 CYN (not shown). While *Flavobacterium johnsoniae* contains four FKBPs in addition to three CFBPs (Table 2), in the same genus, *F. daejeonense* and *F. psychrophilum* each contains just one CFBP, and no FKBP or CYN (not shown). In all cases, the paralogs of CYN and FKBP are nonidentical and also different from the CYN and FKBP sequences of the DFPPI [17,18]. As a rule, organisms that have FCBP do not have single FKBPs. A rare exception is the oomycetes, *Phytophthora infestans*, which harbors a single FKBP-3TPR-CYN (XP_002906896.1) as well as several FKBP sequences, some with TPR domains at the C-terminal side of the FKBP domain (e.g., XP_002901647.1). However, in such cases, the FKBP and the TPR domains are nonidentical in sequence to their counterparts in the FCBP sequence.

In summary, the origin of the nonidentical DFPPI and single PPI paralogs in a single organism, and their apparently random occurrence in a few species, cannot be explained by simple gene duplication, gene fusion, or gene transfer [17,18].

Our TCC model offers an advantage of co-folding to the DFPPI organisms under extreme conditions, but many other organisms that apparently thrive equally well in comparable habitats, contain single PPIs only, and no DFPPI. A glaring example is the Archea, which are patently devoid of DFPPIs, but many of which live in exotic environments. Phylogenetically, we noted only two consistent generalizations [17], which I will reiterate here: (1) the DFPPIs are strictly monocellular, CFBP occurring in bacteria only, and FCBP, in monocellular eukaryotes only, and (2) essentially all FCBPs contain a 3TPR linker in the configuration FKBP-3TPR-CYN, whereas the CFBPs do not have TPR. Based on these patterns, it was proposed that each class of DFPPI initially appeared through a random recombination event, and then remain selected due to the survival advantage discussed earlier. At different points thereafter, they were shared with closely related organisms through horizontal DNA transfer, followed by sequence changes for optimal functioning in the new host. The DFPPI organisms, spread over separate branches of the phylogenetic tree, therefore, may define an Operational Taxonomic Unit or OTU [17].

## 7. Future Directions of Dual-Family Peptidylprolyl Isomerase Research

As said before, an understanding of the DFPPIs will urgently need specific experimental data. Here, I offer possible future directions of research that should shed new light in this area. First of all, a key question is whether both modules possess PPIase enzymatic activity in all DFPPIs, since so far this has only been demonstrated for TgFCBP [16]. A related question is whether the two PPIase modules are functionally identical or whether they exhbit substrate preferences, distinct from each other. Obviously, we need to identify their folding clients, which is not a trivial task, since many proteins are likely to be folded. These studies may involve the use of established protein-protein interaction techniques, such as cross-linking in vivo, followed by co-immunoprecipitation and mass-spectrometry. Second, the phenotype of DFPPI mutant organisms needs to be characterized in detail. Molecular genetic techniques exist for a few DFPPI organisms such as *F. johnsoniae* and several Apicomplexan parasites, most notably, *T. gondii*. The mutant may exhibit loss of viability or motility, but only in more restrictive environments, such as higher temperatures. The phenotype may accompany simultaneous loss of binding to multiple clients, which will validate the physiological relevance of the interaction. One can then compare the interactome of one DFPPI with that of an ortholog in a different species, which may illuminate phylogenetic commonality and differences of their client repertoire. Identification of multi-subunit clients, such as flagellar motor complexes and transporters, and localization at the membrane, will confirm and extend our findings in *T. gondii* and authenticate the TCC model. Lastly, attempts should be made to co-crystallize DFPPI in complex with peptides from client protein sequences, since such binding may stabilize a single conformation in the DFPPI structural ensemble, allowing the formation of crystals [20]. Intrinsically disordered regions are receiving renewed attention as modules of protein-protein interaction and as druggable targets [21,54]. Incidentally, the CFBPs constitute the first examples of a PPIase family that possess intrinsic disorder, and thus, can be explored as targets of small molecules to inhibit the growth of these DFPPI organisms, most of which are serious pathogens of human (e.g., Toxoplasmosis by *T. gondii*, syphilis by *Treponema pallidum*, periodontal disease by *Treponema denticola*, and Lyme disease by *Borrelia* sp.), plants and various other organisms [17].

## Figures and Tables

**Table 1:** List of organism families containing dual-family peptidylprolyl isomerase (DFPPI) genes ^1^.

Organism (Kingdom/Family)	Number of DFPPIs Found
**Bacteria (prokaryotes); total CFBP**	**278**
Bacteroides (Flavobacteria)	235
Spirochetes	26
Proteobacteria	7
Others	10
**Eukarya (all Monocellular); total FCBP**	**44**
Apicomplexa	13
Oomycetes	11
Ciliophora	8
Diatom	4
Dinoflagellates	2
Others	6

^1^ All DFPPI organisms are monocellular; while CFBPs occur in prokaryotes, the FCBPs are found in eukaryotes. I suggest that DFPPIs are specialized bifunctional chaperones that use their flexible interdomain linker to associate with large polypeptides or multisubunit megacomplexes to promote simultaneous folding or renaturation of two clients in proximity, essential in stressful and denaturing environments. Analysis of sequence homology and predicted 3D structures of the FKBP and CYN domains as well as the TPR linkers (Figure 3) upheld the modular nature of the DFPPIs and revealed the uniqueness of their TPR domain. The CFBP and FCBP genes appear to have evolved in parallel pathways with no obvious single common ancestor. The occurrence of both types of DFPPI in multiple unrelated phylogenetic clades supported their selection in metabolic and environmental niche roles rather than a traditional taxonomic relationship. Nonetheless, organisms with these rare immunophilins may define an Operational Taxonomic Unit (OTU), bound by the commonality of chaperone function.

**Table 2:** Examples of co-existence of DFPPI and PPI paralogs ^a^.

Organism	DFPPI/Single PPI	GenBank #	Size (Amino Acids)
***Toxoplasma gondii***			
	FCBP	AAX51680.1	521 [16]
	CYN	XP_018637313.1	106
	CYN	XP_018636397.1	165
	CYN	XP_002369951.1	179 [53]
	CYN	XP_002365722.1	195
	CYN	XP_002369214.1	211
	CYN	XP_002367963.2	237
	CYN	XP_018637703.1	283
	CYN	XP_002365354.1	311
	CYN	XP_002367801.1	348
	CYN	XP_002366733.1	575
	CYN	XP_002367918.1	587
	CYN	XP_002366408.1	592
	CYN	XP_002370366.1	612
	CYN	XP_002369921.1	764
***Flavobacterium johnsoniae***			
	CFBP	WP_012024258.1	310
	CFBP	WP_012024434.1	357
	CFBP	WP_012024433.1	372
	FKBP	WP_071634832.1	151
	FKBP	WP_066033786.1	157
	FKBP	WP_071638913.1	208
	FKBP	WP_066033733.1	380

^a^ Only two organisms are shown to conserve space, but there are several other DFPPI organisms that also contain single CYN and/or FKBP genes. Where a reference is cited [16,53], the protein was experimentally validated and studied.

**Figure A1:**
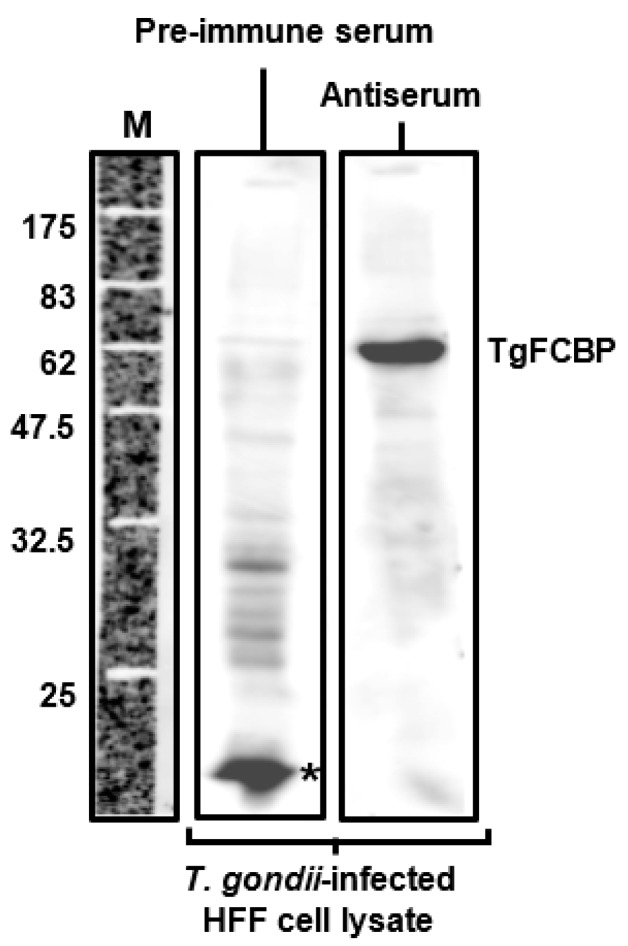
Specificity of the *T. gondii* FCBP (TgFCBP or TgFCBP57) peptide antibody. Immunoblot was performed as described above. Peptide serum, labeled as “antiserum”, detected the 57 kDa TgFCBP band, not detected by the pre-immune serum. Molecular weight markers (M) are indicated. The asterisked band reacted with pre-immune serum but not with the antiserum.

## Appendix A

The antibody used in Figure 5 was tested by immunoblot, shown below (Figure A1). Total cell extract (40 μg protein) from *T. gondii*-infected (18 h post-infection at multiplicity of infection 1.0) human foreskin fibroblasts (HFF) were analyzed by standard sodium dodecyl sulfate-polyacrylamide gel electrophoresis (SDS-PAGE); immunoblotting was performed with a 1:1000 dilution of mouse antiserum, raised against a TgFCBP peptide, described before [16], and horse radish peroxidase-conjugated secondary antibody. The blot was developed by enhanced chemiluminescence (ECL), and the image was captured in LI-COR Odyssey imager (LI-COR Biosciences, Lincoln, NE, USA).
